# Data sharing governance in sub-Saharan Africa during public health emergencies: Gaps and guidance

**DOI:** 10.17159/sajs.2022/13892

**Published:** 2022-10-26

**Authors:** Dirk Brand, Jerome A. Singh, Annelize G. Nienaber McKay, Nezerith Cengiz, Keymanthri Moodley

**Affiliations:** 1 School of Public Leadership, Stellenbosch University, Stellenbosch, South Africa; 2 School of Law, University of KwaZulu-Natal, Durban, South Africa; 3 Division of Law, Abertay University, Dundee, Scotland, United Kingdom; 4 Department of Public Law, University of Pretoria, Pretoria, South Africa; 5 Centre for Medical Ethics and Law, Stellenbosch University, Stellenbosch, South Africa

**Keywords:** data transfer regulations, data sharing, public health, sub-Saharan Africa

## Abstract

While the COVID-19 pandemic has captured the attention of the global community since the end of 2019, deadly health pandemics are not new to Africa. Tuberculosis (TB), malaria and human immunodeficiency virus (HIV) count amongst other serious diseases that have had a catastrophic impact on the African continent. Effective responses to such pandemics require high-quality, comprehensive data sets that can inform policymaking and enhance healthcare decision-making. While data is driving the information economy in the 21st century, the scarcity in Africa of carefully curated, large epidemiologic data sources and analytical capacity to rapidly identify and understand emerging infectious diseases poses a major challenge to mounting a time-sensitive response to unfolding pandemics. Data access, sharing and transfer between countries are crucial to effectively managing current and future health pandemics. Data access and sharing, however, raises questions about personal privacy, the adequacy of governance mechanisms to regulate cross-border data flows, and ethical issues relating to the collection and use of personal data in the interests of public health. Sub-Saharan Africa’s most research-intensive countries are characterised by diverse data management and privacy governance frameworks. Such regional variance can impede time-sensitive data sharing and highlights the need for urgent governance reforms to facilitate effective decision-making in response to rapidly evolving public health threats.

## Introduction

The collation, storage, and sharing of personal data are becoming increasingly important in public health research and surveillance. Balancing data protection and privacy concerns with data sharing and open science is a persistent challenge. The COVID-19 pandemic has highlighted the importance of data sharing in the public interest.^1,2^

While the world has afforded significant attention to the COVID-19 pandemic since the end of 2019, Africa has historically been the perennial epicentre of some of the world’s deadliest diseases, including tuberculosis, malaria, and human immunodeficiency virus (HIV). The clinical and public health management of these pandemics requires the collation, storage, and use of personal and aggregated health data to inform clinical decision-making, scientific endeavours, and policymaking. The COVID-19 pandemic has demonstrated the need for transparency – not just to keep the public informed about the nature and spread of the disease, but also to enable citizens to hold governments accountable for the extraordinary measures they have adopted to manage the pandemic. Seen in this light, data sharing is also in the public interest as the guidance and directives the public is expected to follow, should be evidence-based.

The rapid global spread of COVID-19 has necessitated greater international cooperation. Although optimised data collection at a national level is important to strengthen the domestic response to COVID-19, regional and international cooperation in accessing and using such data to inform policymaking beyond the data source setting is equally important due to the global impact of the disease.^3,4^ Not surprisingly, the World Health Organization (WHO) has repeatedly stressed the need for cooperation between countries to end the COVID-19 pandemic.^5^

At a very basic level, there should be appropriate infrastructure in a country for the effective collection of personal information of data subjects, and the storage thereof. Moreover, a country should possess analytical capacity to understand the implications of the data. Limited Internet access, poor quality data sources, and inadequate analytical capacity characterise many African settings.^6^ In the context of COVID-19, such shortcomings contributed to critical knowledge gaps, such as the impact of COVID-19 on African children.^7^ The scale of the current COVID-19 pandemic has underscored the need for appropriate data collation, storage, and analysis globally to strengthen evidence-based approaches to managing the pandemic.^6,8^ Although many African countries have promulgated data protection legislation, the practical gap between the availability of data sources and analytical capacity is of serious concern. While such gaps negatively impact local and national efforts to fight the pandemic, they also underscore the global inequity regarding the resources and ability to manage health pandemics.^9,10^

## Data requirements to manage COVID-19 and other health pandemics

The lack of harmonised data sets and information systems poses a major barrier to the effective management of public health emergencies. Moreover, time-sensitive access to data may not always be possible.^11,12^ Access to large, high-quality data sets is also crucial to the development of artificial intelligence (AI) models, which can help optimise predictive decision-making in health pandemics.^13^ One of the basic building blocks in managing health pandemics at a country level is the development of national health information systems that include both public and private sources of health data.

Broad categories of data are required to effectively respond to pandemics. Primary data sources include, but are not limited to, mobility surveillance data, incidence, hospitalisation, recovery, and mortality data aggregated by amongst other factors, age, sex, comorbidity, and vaccination status. Epidemiological data and omics research data are, from a research perspective, critical data sources that support an evidence-based approach to managing a pandemic.^14^ An inability to gain timely access to data, trust deficits, diverse data sources and diverse data needs are amongst the factors that can hinder an effective response to a pandemic (Box 1).^15–18^ To overcome such barriers, the characterisation and formats of various health data sources should be harmonised and standardised to facilitate sharing amongst relevant stakeholders, such as researchers, public health officials, and international agencies.

In considering data sharing needs during a pandemic, it is important to reflect on how ‘open data’ could facilitate access to, and use of, health data. The scale of the COVID-19 pandemic and the need to have time-sensitive epidemiological data publicly accessible on a daily basis has boosted support for open data. An important motivating factor for open data is the opportunity such data creates for a diverse group of stakeholders, including researchers and software developers, to analyse the data and generate new insights and applications.^19^ The internationally renowned COVID-19 global dashboard created by the Johns Hopkins University^20^ and other similar country-level dashboards that are updated daily, count as examples of how open data can add value to the management of pandemics. Open data, however, also present challenges and potential risks. Some commentators note that government efforts to provide and maintain open data are costly and require considerable expertise, which is not universally available.^19^

Data quality, access to primary data and completeness of data are additional challenges to open data. Data access governance, intellectual property rights and privacy considerations also merit noting.^21^ When open data applications are created, data anonymisation is usually part of that creation process. However, privacy concerns could arise if there remains a possibility to de-anonymise personal data, notwithstanding technological tools, such as differential privacy used in machine learning modelling, which could be used to respond to such privacy concerns. Despite such potential risks and challenges, the benefit of timely open data during a pandemic is clear. The COVID-19 pandemic has demonstrated the key role that bioinformaticians and epidemiologists can play in collating and analysing data sets to rapidly inform pandemic decision-making.^22^ Such contributions would not be possible without the collection of, and time-sensitive access to, large, high-quality data sets.

## Initiatives to facilitate cross-border data sharing in Africa

In 2002, the WHO published an Integrated Disease Surveillance and Response (IDSR) for WHO’s African region, which was widely adopted by African member states.^23^ The African Union (AU) is similarly leading an effort to harmonise statistical data sets to facilitate and foster data sharing in Africa.^24^ In 2018, the Africa Centres for Disease Control (Africa CDC), which serves as a specialised technical institution of the AU, published a Framework for Event-based Surveillance, which is intended to complement and enhance the implementation of IDSR.^25^ In publishing the third edition of its IDSR Guidelines in 2019, the WHO conceded that progress towards a coordinated, integrated surveillance system in Africa has been mixed.^23^ In recognition thereof, the Africa CDC has stepped up its efforts to support Member States to develop and establish high-quality public health information and technology systems.

One of the Africa CDC’s flagship initiatives involves the development of a continental wide public health information system platform by linking public health institutes in each country through a wide area network managed by the Africa CDC.^26^ The strategic objective of this initiative includes, amongst others, enhancing secure electronic transmission of relevant data and reports, facilitating the development and promotion of network domains, and adopting informatics guidelines and standards to enable interconnectivity and electronic transmission of data and information among Africa CDC institutes.^26^

Similarly, the Africa CDC has also established the Regional Integrated Surveillance and Laboratory Network (RISLNET) initiative, which aims to establish an integrated electronic network of regional surveillance platforms by leveraging existing regional public health assets, such as the surveillance and laboratory networks operated by public agencies, private organisations, foundations, and universities in eastern and southern Africa.^27^ Data from RISLNET is intended to inform, amongst others, the Extension for Community Healthcare Outcomes (ECHO) platform, which aims to share critical, timely, lifesaving information and data with healthcare workers at different geographical locations.^28^ Digital disease surveillance is described as ‘the aggregation and analysis of data available on the internet, such as search engines, social media, and mobile phones, and not directly associated with patient illnesses or medical encounters’^29^. In recognition of its potential to contribute to disease surveillance, the Africa CDC has established a pilot digital disease surveillance programme aimed at conducting real-time surveillance of infectious diseases in Africa by monitoring social media and building capacity in ‘Big Data’ approaches for outbreak prediction, analysis, and prevention.^29^ Such ambitious regional initiatives speak to the need for time-sensitive cross-border data sharing but also raise privacy concerns.

## Data stewardship: Ethics and governance considerations

The Open Data Institute characterises data sharing in three aspects: (1) stewardship of data (collection, maintenance, sharing); (2) creating information from the data (analysis, insights); and (3) making informed decisions utilising data from different sources.^30^ Various technical, motivational, economic, political, legal, and ethical barriers can negatively impact on public health data sharing initiatives.^31^ Such barriers include a lack of resources in the public sector (economic barrier), ownership and copyright (legal barrier) and lack of reciprocity in data sharing practices (ethical barrier). The COVID-19 pandemic has underscored the need to develop a universal system or standard for collating and sharing data, including research outputs based on such data.^32–34^ This universality is especially crucial during the initial stages of an emerging pandemic when information about the nature and spread of a disease should be shared internationally as soon as possible, as well as later, when coordinated evidence-based efforts to fight the pandemic are important. In 2022, the OECD published recommendations on enhancing access to and sharing of data^12^, which include the principles and guidelines captured in Box 2. In a research report^24^ that focuses on data sharing to enhance public health in Africa, equitable, ethical, and efficient data sharing constitute key principles.

The United Kingdom (UK) government has followed a practical approach in devising its Data Ethics Framework (DEF), which offers guidance for responsible data use in the public sector.^35^ The DEF posits three principles, namely transparency, accountability, and fairness. In this context, transparency means that data processes are open to inspection and that information about a project must be published in an understandable and accessible format. Adherence to accountability means that governance and oversight mechanisms are in place and implemented effectively. To mitigate any bias or discrimination in the capturing, sharing and use of data, the principle of fairness must apply, that is, there must be respect for the dignity of individuals and an aim to deliver fair and non-discriminatory outcomes. Despite being UK-focused, DEF’s guidance principles are universally relevant and should be considered in the context of cross-border data transfers between African countries. Transparency, accountability, and fairness are also legal principles underpinning administrative and constitutional law. Although they are not necessarily included in data protection legislation, they are still important in the context of responsible data use and should guide the drafting of data transfer agreements.

Some commentators have argued for a wide approach to drafting a data transfer agreement in a research context, suggesting that it should include, amongst others, provisions on ethical considerations including ethical approval, and benefit sharing.^36^ Novel ideas such as benefit sharing could be considered from a research ethics perspective, but the *Protection of Personal Information Act* (POPIA) and other data protection legislation are aimed at protecting personal information and not aimed at research ethics per se. Although the detail of a data transfer agreement is context specific, and anchored in the relevant data protection legislation, any data transfer agreement in a research context should, at minimum, include the elements indicated in Box 3. In some settings, national data protection authorities may need to prospectively approve cross-border transfers involving personal data.

The Research Data Alliance (RDA) COVID-19 Working Group has published a set of recommendations and guidelines relating to the collection and sharing of data in the context of COVID-19.^11,12^ The work of the RDA COVID-19 Working Group is divided into four categories, namely clinical research, omics, epidemiology, and social sciences, all of which contribute to the multidisciplinary nature of managing a pandemic. In all these areas there is a need for more data sharing, but often a lack of proper data sharing or data transfer agreements negatively impacts the analytical work as well as the policy responses as part of the management of a pandemic.

## Africa’s data sharing regulatory landscape

Data protection laws provide the legal framework for the collection, access to and sharing of data, as well as the cross-border transfer of data. Many African countries have embarked on initiatives to regulate data protection. In the fields of public health, environmental and occupational health, South Africa, Nigeria, Kenya, Ethiopia and Uganda^37^ rank respectively as the most research-intense countries in sub-Saharan Africa by research output^32–34^. It is thus apt to briefly consider how these settings manage cross-border data transfers.

Table 1 categorises the rigour of national data protection laws regarding cross-border transfer of personal data.^38^ Table 1 is not aimed at providing a strict overall categorisation of various data protection laws, but rather, is focused on the scope of legal protection afforded to data subjects in relation to the cross-border transfer of their personal data. Countries with *stringent* rules require notification of, or approval by, a relevant data protection authority, and/or special conditions (such as proof of appropriate safeguards with respect to the protection and security of personal data), as well as consent from the data subject.

South Africa and Kenya count amongst countries that could be described as providing stringent data export protection to data subjects. For example, *Kenya’s Data Protection Act of 2019* complies with the European Union (EU) legal standards, which are generally regarded as being stringent in nature. For data to be transferred out of Kenya, the data processor must verify to the data commissioner that the third-party recipient’s jurisdiction is bound by appropriate safeguards for the security and protection of the data. It is also important that the data transfer be purposeful, such as being necessary for the conclusion or performance of a contract or legal claim, and the public or data subjects’ interests. In addition, consent from the data subject is also required for cross-border data transfers.^38^

Countries falling in the *moderate* category allow for more than one possible legal ground to permit data export, such as consent of the data subject, but do not require notification or approval by the data protection authority. Nigeria counts amongst countries providing moderate data export protection to data subjects as the country’s data protection law does not require third-party recipients of data to be bound by adequate data protection law, agreements, or corporate rules if the data subject provides consent after being informed of possible risks of inadequate data protection or if the transfer meets a certain exception. One example of such exception is the public’s or data subject’s interest. Beyond obtaining consent from data subjects for data transfers, the Nigeria Data Protection Regulation 2019 requires the National Information Technology Development Agency (NITDA) or Honourable Attorney General of the Federation (HAGF) to ensure that the third-party recipients of the transferred data have adequate data protection standards in place.^38^

Ghana’s data protection legislation does not contain any provisions pertaining to cross-border transfer of personal information^39^ and could thus be described as providing *inadequate* protection to data subjects in relation to the export of their personal data.

The diverse legal landscape governing data sharing in sub-Saharan Africa – including the stringency of data export provisions – highlights that cross-border data transfers will have to be evaluated on a case-by-case basis as there is no uniform law across the continent akin to the General Data Protection Regulation (GDPR 2018), which constitutes a common legal framework for all EU Member States. Although the AU Commission is developing a data policy framework for Africa to harness digital technologies and innovation in an attempt to bridge the digital divide, this process is ongoing and will take time to implement.^16^ Further, the AU Convention on Cyber Security and Personal Data (2014) has been ratified by only 13 AU Member States.^40^

It is important to note that in addition to specific data protection legislation, 29 sub-Saharan countries have published some form of research ethics guidance regarding the collection and use of human biological specimens, which are essential in national health systems and important to consider in international research cooperation.^41^ These countries are Benin, Botswana, Burkina Faso, Cameroon, Democratic Republic of the Congo, Equatorial Guinea, Ethiopia, Gabon, The Gambia, Ghana, Guinea, Kenya, Lesotho, Liberia, Madagascar, Malawi, Mali, Mauritius, Mozambique, Nigeria, Rwanda, Senegal, Sierra Leone, South Africa, Sudan, Tanzania, Uganda, Zambia, and Zimbabwe.^41^

These guidance documents do not specifically govern data sharing. In South Africa, the Academy of Science of South Africa (ASSAf) – a statutory body and the country’s apex science advisory body, has developed a privacy Code of Conduct for Research. The Code is intended to provide guidance to the research community about the use of data in research and is binding.^42^ The Code reiterates the country’s legislative stance on the transfers of personal information outside the country.

It is evident from the above that data sharing governance varies across Africa. Adequacy of legal protection for cross-border transfers is of particular importance when scientists and other stakeholders in different countries need to share data to effectively respond to a rapidly evolving pandemic. Cross-border data transfers of personal information may only take place if certain requirements are met in accordance with the applicable local legislation. A common approach found in various data protection laws includes the imposition of certain conditions, as indicated in Box 4.

Data transfer agreements can facilitate the cross-border transfer of data and catalyse international research collaboration.^17^ A lack of pre-approved data sharing agreements and archaic health data systems count amongst the critical shortcomings of the global community’s response to the COVID-19 pandemic.^17,26^ To avoid similar shortcomings in the future, relevant authorities should develop standardised tools and templates for international research collaboration. The development of such tools and templates should be based on sound governance and ethics principles. Additional factors to consider in the processing of personal information are outlined in Box 5.

## Conclusion and recommendations

This article highlights the factors that impact data sharing in sub-Saharan Africa, especially in the context of managing health pandemics. The COVID-19 pandemic has underscored the need for a reliable and accessible data ecosystem that could inform the management of public health threats. The combined effect of diverse limitations or barriers to data sharing in public health necessitates more dedicated continental and international cooperation as well as the development of standard formats for health data. Harmonised data sources and their integration into national health information systems will create a comprehensive data set that includes epidemiologic, clinical as well as behavioural data relating to public health emergencies. Such a holistic approach to data management should underpin evidence-based decision-making. The principles of transparency, fairness and accountability should underpin the development of a reliable and accessible data ecosystem. To facilitate cross-border data transfers involving personal data, standard contractual provisions and templates for cross-border data transfers should be developed by data protection authorities in Africa. This will facilitate not just scientific cooperation between countries, but also an integrated cross-border approach to the management of future pandemics.

## Figures and Tables

**Table 1:** Data protection laws, regarding cross-border transfer of personal data, of sub-Saharan African countries ranked by research output in “Public Health, Environment, and Occupational Health”^37^

Rank and country	Legal requirements	Legislation	Data export protection classification
South Africa	A responsible party may only transfer personal data outside South Africa if the recipient is subject to a law, binding corporate rules or binding agreement that provides adequate protection; or the data subject consents to the transfer; or the transfer is necessary in terms of the provisions of the Act.	Section 72 of the *Protection of Personal Information Act, 4 of 2013*	Strict
Nigeria	Cross-border transfer of personal data is subject to authorisation by the Attorney General or the National Information Technology Development Agency (NITDA) based on an adequate level of protection. In the absence of authorisation by the Attorney General or the NITDA, personal data transfer may only take place if the data subject gives consent, or the data transfer is necessary in terms of the Regulation.	Reg. 2.11 and 2.12 of the Nigeria Data Protection Regulation, 2019	Moderate
Kenya	Data transfer is allowed only if there is proof of adequate data protection safeguards or consent from the data subject. The data controller or data processor must provide proof of appropriate safeguards to the Data Commissioner. The data transfer must be necessary in terms of the Act.	Section 25(h) 48 of the *Data Protection Act, No. 24 of 2019* (Kenya)	Strict
Ethiopia	Cross-border data transfer may only take place subject to an adequate level of data protection in the recipient country. The data controller or data processor must provide proof to the Data Protection Commission of an appropriate level of protection, or the data subject must give consent to the proposed transfer, or the transfer must be necessary, or the transfer must be made from a register and intended to provide information to the public.	Sections 27–30 of the Draft Proclamation to Provide for Personal Data Protection, 2021 (Ethiopia)	Strict
Uganda	The data processor or data controller must ensure that there are adequate measures in place for the protection of personal data, or the data subject must provide consent.	Section 19 of the *Data Protection and Privacy Act, 2019* (Uganda)	Strict

## Significance:

We explore governance considerations that ought to apply to the collection, transfer, and use of data in public health emergencies. Specifically, we provide an overview of the prevailing data sharing governance landscape in selected African countries. In doing so, we identify limitations and gaps that impede effective data collation, sharing and analysis. This work could find utility amongst a range of stakeholders, including bioinformaticians, epidemiologists, artificial intelligence coders, and government decision-makers. While this work focuses primarily on an African context, the issues explored are of universal concern and therefore of relevance to a broader international audience.

## Box 1: Key limitations in data sharing

- Timely access to data during a public health emergency is critical to gain detailed knowledge of a pandemic that could assist in developing effective public health responses. Many low- and middle-income countries have limited capacity to undertake epidemiological, clinical, and other research, thus negatively impacting on the ability to respond effectively to public health emergencies.
- Data sharing, including cross-border transfer of data, is limited due to a lack of trust about the future use of the data, insufficient data transfer regimes or the academic competition to be the first to publish results of scientific research and thus unwillingness to share research data.
- Data needs of different stakeholders differ, which makes a common approach to data sharing difficult.^17^
- There is a variety of types of data collected and used in the context of public health, which complicates effective data sharing practices.
- In cases where data quality is questionable or the data is incomplete, the scope of using the data in the management of a health pandemic is limited.
- Colonial legacies and social and economic inequalities could have a negative impact on international cooperation to share data due to questions about trust between international partners.^18^
- A lack of standardisation of types of data limits international cooperation.^15^

## Box 2: Recommendations of the Council on Enhancing Access to and Sharing of Data in 2022

- Promote trustworthiness of the data ecosystem.
- Enhance transparency of data access and sharing arrangements.
- Incentivise data access and sharing.
- Foster effective and responsible data access, sharing and use across society.

## Box 3: Minimum requirements for a data transfer agreement

- Responsibilities of the provider and recipient of the data
- Purpose of the use of the data
- Description of the data
- Time period of the agreement
- Access to the data (e.g. in a research context there could be various people in the working environment of the recipient who need access to the data)
- Confirmation of adherence to all the legal requirements for lawful processing of data
- Publication of the research results based on the transferred data
- Ethical clearance for the use of the data in the research
- Dispute resolution provisions

## Box 4: Common conditions enforced in various data protection laws

- The third-party recipient of the data is subject to a law, binding corporate rules or a binding agreement, which provides adequate protection.
- The data subject gives consent for the cross-border transfer.
- The transfer is necessary for the performance of a contract between the data subject and the responsible party/controller.
- The transfer is necessary for the conclusion or performance of a contract in the interest of the data subject between the responsible party and a third party.
- The transfer is for the benefit of the data subject.

## Box 5: Factors to consider in the processing of personal information^18^

- The need for a time limit for the retention of the data
- Clarity and a sound legal basis for the purpose of the data processing
- Proportionality of the measures taken in processing the personal data
- Transparency and explainability
- Accountability
- Integration of privacy by design
- Realisation of data protection impact assessments
